# ADVANTAGE: Advanced discovery of visceral analgesics by neuroimmune targets and the genetics of extreme human phenotype, a study protocol

**DOI:** 10.1371/journal.pone.0350169

**Published:** 2026-05-21

**Authors:** Marco Vinicio Alban-Paccha, Nicholas Shenker, Jimena Teran-Perez, Andrew W. Horne, George G. Malliaras, Christopher Geoffrey Woods, on behalf of the ADVANTAGE Consortium

**Affiliations:** 1 Department of Medicine, University of Cambridge, Cambridge, United Kingdom; 2 Electrical Engineering Division, Department of Engineering, University of Cambridge, Cambridge, United Kingdom; 3 Rheumatology Research Unit, Addenbrooke’s Hospital, Cambridge, United Kingdom; 4 Centre for Reproductive Health, Institute of Regeneration and Repair, The University of Edinburgh, Edinburgh, United Kingdom; 5 Department of Medical Genetics, Cambridge Institute for Medical Research, University of Cambridge, Cambridge, United Kingdom; Children Hospital Lahore: University of Child Health Sciences, PAKISTAN

## Abstract

Chronic visceral pain affects over 20% of adults globally but remains poorly understood, significantly impacting quality of life and healthcare costs. Limited understanding and diagnostic misconceptions hinder effective management, particularly during acute pain flares. This study aims to clarify underlying mechanisms and improve clinical management by combining detailed phenotyping, genetic analysis, immunological profiling, pain mapping, and wearable sensor data in three cohorts: Extreme Visceral Pain, Lack of Visceral Pain, and Healthy Controls. Participants with diverse visceral conditions, such as polycystic kidney disease, inflammatory bowel disease, chronic pancreatitis, endometriosis, painful bladder syndrome, vaginal mesh complications, and fibromyalgia, are recruited via clinical referrals from NHS Cambridge University Hospitals and NHS Lothian. Healthy control volunteers are recruited locally. Data collection involves daily pain ratings captured through a mobile app, wearable physiological monitoring, quantitative sensory testing, detailed medical and lifestyle questionnaires, and bio-sample analyses (genetic variants, autoantibodies). The primary confirmatory outcome evaluates the correlation between wearable sensor parameters and self-reported visceral pain intensity. Genetic analyses, including functional SNP allele discovery and Mendelian gene effect analysis, and immunological profiling will explore underlying biological mechanisms. Challenges anticipated include potential compliance and technical difficulties in remote data collection, potentially affecting data quality. Findings will be disseminated widely, aiming to refine diagnostic tools and inform treatment strategies, ultimately enhancing patient care and outcomes in chronic visceral pain management.

## Introduction

Chronic visceral pain originating from the chest, abdomen and pelvis organs, is estimated to affect up to 25% of the adult world population [1], and imposes a significant economic burden on global healthcare systems [2], with annual costs exceeding $50 billion in the US for chronic visceral pain and £100 million in the UK for abdominal pain alone [3]. Beyond the economic toll, this kind of pain disrupts essential daily activities such as urination, defecation, and sexual intercourse. Unpredictable episodes of severe visceral pain, known as “flares”, often require hospitalization and are a common challenge across various visceral pain conditions [4,5].

Despite its prevalence and impact, our scientific understanding of visceral pain remains surprisingly limited. Psychological factors are known to play a role in maintaining chronic pelvic or urogenital pain, with stress being a recognized aggravator of it. Yet, these biological associations are poorly understood and are often misinterpreted, which hinders proper diagnosis and treatment. Equally unclear is the relationship between the level of pain experienced by patients and the severity of their underlying visceral disorder. Pain often persists even when the visceral pathology is adequately treated, such as during remission in conditions like ulcerative colitis [6] or following surgery for endometriosis [7].

There are significant gaps in our knowledge of basic visceral pain mechanisms. For example, we lack detailed information on the sensory innervation of key tissues like the kidney and uterus, and the roles of the vagus and peripheral nerves in visceral pain remain unclear. Currently, there are no specific analgesics for visceral pain; the most prescribed drugs are antispasmodics, while conventional analgesics like opioids and nonsteroidal anti-inflammatory drugs (NSAIDs) are associated with problematic side effects. Psychological approaches, although beneficial, are typically designed for musculoskeletal pain and do not adequately address the unique concerns of visceral pain patients [8].

Most diagnoses of visceral pain are based on the presumed origin of the pain within specific organs. However, there is a scarcity of data on the physiological and behavioural changes that accompany visceral pain episodes, particularly in natural, free-living conditions. Currently available pain assessment tools designed for musculoskeletal and neuropathic pain fail to adequately capture the complexity, variability, and acute episodic nature of visceral pain, particularly during intimate functions or acute flares that often lead to emergency department visits despite being brief and difficult to study in controlled settings [3,9]. In clinical practice, the challenge arises when diagnostic investigations fail to reveal an organic cause for pain perceived to originate from the viscera. Syndromal disorders, named after the presumed pain origin, often dissociate pain from the underlying organ pathology [10], such as persistent pain in inflammatory bowel disease during remission or pain in polycystic kidney disease that does not correlate with kidney size.

The relationship of visceral pain with broader pain syndromes, such as fibromyalgia, is also poorly understood. For some patients, managing visceral pain can alleviate fibromyalgia symptoms, yet not all fibromyalgia patients experience visceral pain, and the reasons for this disparity are unclear. Similarly, conditions like migraine, myofascial syndromes, and complex regional pain syndromes have been linked to visceral pain, but the nature and meaning of visceral pain in these contexts are not well quantified [11,12].

The development of digital tools, wearable sensors, and home point-of-care testing offers new opportunities to study visceral pain in real-world settings. These technologies enable continuous monitoring of pain and related physiological parameters, providing insights that were previously unattainable in hospital or laboratory settings [13,14]. By capturing data in everyday environments, wearable sensors can track fluctuations in visceral pain intensity and correlate them with physical activities, posture, and physiological changes such as heart rate and respiratory patterns. This allows for a more comprehensive understanding of pain triggers and patterns, offering the potential to personalize pain management strategies. The continuous nature of this data collection also facilitates early detection of visceral pain flares, enabling timely interventions and reducing the need for hospital visits.

Visceral pain may also result from unidentified pathophysiological processes within the viscera or from abnormal sensitization of the ‘pain pathway’ within the sensory nervous system, a complex signalling network relaying information from peripheral tissues via the spinal cord to the brain. Thus, visceral pain is assumed to originate from the activation of nociceptors, specialised nerve fibres that sense pain in thoracic, pelvic, or abdominal organs. Sustained nociceptive input can then lead to neuroplastic changes in the central nervous system (CNS), altering neural circuits and potentially increasing pain sensitivity and persistence over time [15]. Conversely, the role of other peripheral nerves, such as the sympathetic and vagus nerves, in detecting, localizing, and perpetuating visceral pain remain largely unknown.

Most current drugs used to treat visceral pain primarily target neuroplastic changes in the CNS, and many are even prescribed “off-label” for this purpose. However, new evidence suggests these treatments may be ineffective or harmful in some conditions, such as endometriosis [16] or narcotic bowel syndrome [17]. This has led to growing interest in developing medications that target visceral nociceptors [18], with the hope of identifying effective peripheral treatments that have reduced side effects and thus improved patient outcomes.

This study represents a novel integration of real-world digital phenotyping with multi-omics approaches to investigate visceral pain. While individual components, such as wearable technology, genetic analysis, and immunological profiling have been applied separately to chronic pain research, this is one of the first comprehensive studies to systematically combine continuous physiological monitoring via wearable sensors with exome sequencing and autoantibody analysis specifically in visceral pain cohorts. This integrated approach enables simultaneous investigation of peripheral and central mechanisms, environmental triggers, and genetic predisposition within naturalistic settings, offering unprecedented opportunities to delineate visceral pain phenotypes and identify novel therapeutic targets distinct from existing musculoskeletal pain frameworks.

### Aims and endpoints

The overarching aim of this study is to deepen our understanding of chronic visceral pain by identifying and evaluating the genetic, molecular, and physiological mechanisms that contribute to this condition. We will examine these mechanisms across different participant groups, including those with extreme visceral pain, those with a lack of expected pain despite visceral disease, and healthy controls.

Our **primary objective** is to look for physiological markers (such as changes in movement, heart rate, and postural patterns) that predict or associate with visceral pain flares by examining data from wearable sensors. This will provide objective, real-time insights into how visceral pain affects day-to-day activities, and will be tested using a pre-specified statistical model and is central to the confirmatory aspect of the study.

In addition, we will undertake a series of **secondary/exploratory objectives**, which include the following key areas of investigation:

1 **Genetic insights:** We aim to identify and characterize the genetic factors that underlie chronic visceral pain in specific participant cohorts, which include individuals with conditions like polycystic kidney disease, inflammatory bowel disease, chronic pancreatitis, endometriosis, and painful bladder syndrome.
2 **Spatial and temporal pain mapping:** We will create detailed maps of where and when individuals experience visceral pain. This includes analysing the frequency and intensity of pain reported by participants using a remote mobile app and wearable sensors that track body movement, posture, heart rate, and breathing.
3 **Biological sample analysis:** We will collect a variety of biological samples—blood, urine, low vaginal swabs, and faeces—from participants to explore pain mediators and modulators. This will include testing human serum in passive transfer experiments using mice to better understand the role of autoantibodies and other pain-related factors [19].
4 **Comparing pain groups:** We will compare participants with high levels of visceral pain, regardless of disease activity, to those with little or no visceral pain despite having active disease. These groups will be contrasted with healthy control subjects to uncover key differences in pain experience and physiology.

The **primary endpoint** will focus on preliminary analysis of the wearable sensor data. We will assess the relationship between wearable sensor parameters (such as heart rate variability, physical activity, and posture) and self-reported pain intensity in participants with chronic visceral pain. We aim to determine whether specific physiological markers—such as body posture— can reliably predict or associate with pain levels. This analysis will provide clinically useful insights into how wearable sensor data can be used to quantify and monitor visceral pain in real-time.

The **secondary endpoints** will focus on exploratory analyses, including:

- Pinpointing the functional pathways involved in the development and progression of chronic visceral pain through genetic analysis. This will help us understand why some individuals develop severe pain while others do not, even with similar disease activity.
- Identifying genetic variants that may regulate visceral pain, particularly in individuals whose pain severity does not align with the activity of their disease. This analysis will explore both known and novel genetic factors that contribute to pain perception.
- Investigating the presence and concentration of autoantibodies to explore their potential role in chronic visceral pain and immune-mediated mechanisms that may contribute to pain persistence.
- Identifying distinct clinical phenotypes of visceral pain using clustering techniques to group participants by shared characteristics, such as disease type, pain severity, and wearable sensor data. These clusters will be analysed for potential genetic or immune-related differences.
- Exploring pain frequency, location (using body maps), and time-series data from self-reported pain ratings to uncover patterns that can help characterize visceral pain flares.

By studying these diverse participant groups and collecting detailed physiological, genetic, and self-reported data, this research aims to provide new insights that could lead to improved diagnostics, treatments, and future studies on chronic visceral pain.

### Methods and analysis

This study is designed to explore chronic visceral pain through the recruitment and analysis of three distinct cohorts: **Extreme Visceral Pain** (individuals with high pain despite low disease activity), **Lack of Visceral Pain** (those with low or no visceral pain despite high disease activity), and **Healthy Controls** (from volunteers). This multi-faceted approach will allow us to examine the complex relationships between pain, disease activity, and biological markers, providing new insights into the mechanisms of chronic visceral pain. Each cohort will undergo tailored assessments, with the primary goal of identifying functional pathways that differentiate pain experiences across these diverse groups.

### Participant recruitment

The study aims to recruit 350 participants across the three cohorts:

- **“Extreme Visceral Pain”:** Participants reporting high pain despite low or no visible disease activity.
- **“Lack of Visceral Pain”:** Participants with active visceral disease but low pain levels.
- **“Healthy Controls”:** Individuals without any chronic visceral pain or relevant disease.

**Fig 1:**
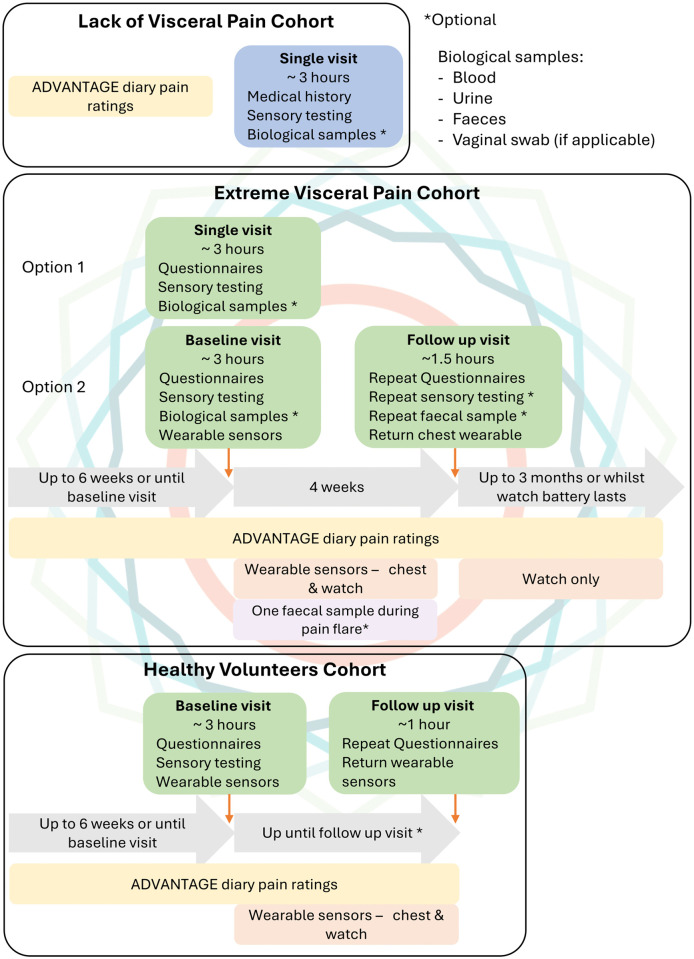
ADVANTAGE Study Protocol Overview Across All Three Cohorts. Comprehensive timeline showing assessment components and participant journey for Extreme Visceral Pain (high pain despite low disease activity), Lack of Visceral Pain (low pain despite high disease activity), and Healthy Controls cohorts. All participants complete baseline visit (~3 hours) with structured clinical interview, validated questionnaires (BPI-SF, McGill, disease-specific scales), quantitative sensory testing (QST: thermal, vibration, pressure pain thresholds), and ADVANTAGE Diary remote mobile app setup for daily pain ratings (0-10 Likert scale). Extreme Visceral Pain and Lack of Visceral Pain cohorts provide biological samples (50 mL blood for DNA/serum, urine, vaginal swabs, faeces). Extreme Visceral Pain and Healthy Controls proceed to 4-week wearable monitoring (chest-worn respiratory sensor, wrist-worn activity/HR sensor) followed by return visit (~1.5 hours) for repeat assessments, device return, and optional bio-sampling.

Participants with a diagnosed disease will be recruited from referrals through clinical advisors, and healthy control participant will be recruited through social media and public advertisements across Cambridge. All participants must be at least 18 years old, able to provide informed consent, and based in the UK. Healthy Controls will be age- and gender-matched with the other two cohorts. After assessing that the individuals can participate in the study and have given consent, each one will be placed in one of the three participant cohorts, and the assessments will be realised according to Fig 1. Eligible participants who have provided consent will be invited to undergo the following assessments:

- Remote mobile app to track pain intensity
- A first onsite visit, consisting of:
  - Health screening through structured interview
  - Questionnaires
  - Physical Examination, including quantitative sensory testing
  - Bio samples collection (not applicable for healthy controls)
  - Training for wearable use (if applicable)
  - Remote mobile app setup to track anxiety, and body position in addition to pain intensity
- Follow-up visit (for participants using wearable sensors only), consisting of:
  - Repeat of procedures from the first onsite visit (sensory testing is optional)
  - Return of wearable

Potential participants in the Extreme Visceral Pain and Lack of Visceral Pain cohorts will be identified by their clinical team based on the inclusion and exclusion criteria outlined in Table 1. Interested individuals can either contact the research team directly or give consent for their clinical team to share their details with researchers. After the initial contact by the clinical team, the research team will follow up to provide more information about the study and ask relevant screening questions, including a pain score if the participant does not have access to the remote mobile app.

**Table 1:** Inclusion and Exclusion Criteria for the Extreme Visceral Pain and Lack of Visceral Pain cohorts. Comprehensive eligibility matrix for the remote mobile app sub-study, wearable sensor sub-study, and bio-sampling sub-study. Common criteria: ≥ 18 years, UK-based, informed consent capable. Sub-study exclusions ensure safety (pacemakers, contact dermatitis), feasibility (travel restrictions), and cohort purity (no confounding pain conditions). Criteria support n = 350 total recruitment with n = 100 wearable sub-study power (80% power).

Sub-study	Inclusion Criteria	Exclusion Criteria
Remote mobile app	Pain, or lack of pain, associated with one or more of the specified visceral diseases or chronic pain conditions as diagnosed by an expert NHS medical professional.	Not able to fulfil the inclusion criteria
	Able to provide informed consent electronically	
	Aged 18 years and over	
	Living in the UK	
Wearable sensor	Able to fulfil the inclusion criteria of the remote mobile app sub-study	Unable to attend or visit the research site.*
		Travel abroad in the plan duration of wearable sub-study
	Have completed and adhered to the procedure in remote mobile app sub-study	
		Unable or unwilling to use body sensor for any reason (e.g. contact dermatitis over wear zone, implanted pacemaker or defibrillator, allergy or hypersensitivity to nickel)
Bio-sampling	Pain associated with one or more of specified visceral diseases or pain condition diagnosis by an NHS medical professional	Unable to attend or visit the research site.*
	Meet further clinical criteria for genetic sampling and analysis (see Supplementary Information)	
	Able to provide informed consent	Not able to fulfil the inclusion criteria
	Aged 18 years and over	
	Lives in the UK	

* Participants are eligible for the study regardless of their location, as long as they are willing and able to travel to the research site.

To enable researchers outside of the clinical care team in the Cambridge site to contact individuals about research opportunities, we will use the existing “consent for contact” system. Through this system, individuals can indicate their interest in research studies via MyChart or an equivalent National Health Service (NHS) health record app, allowing researchers to identify, pre-screen, and contact eligible individuals. Study information will be shared with the potential participants through a Participant Information Sheet (PIS), either in print or electronically (see Supplementary Information for a PIS example).

Potential participants will then be screened and assessed for eligibility. All eligible participants will be offered the option to take part in the remote mobile app sub-study. Those who consent and complete the remote mobile app sub-study may be invited to the research site to join the wearable sub-study. Eligible participants will also be invited to participate in the bio-sampling sub-study.

The Healthy Controls cohort will go through a similar identification by the research team based on the inclusion and exclusion criteria outlined in Table 2. Participants in this cohort will be called to join the study if their demographics align with the current recruitment in the other cohorts by age and gender.

**Table 2:** Inclusion and Exclusion Criteria for the Healthy Controls cohorts. Comprehensive eligibility matrix for the remote mobile app sub-study, and wearable sensor sub-study. Common criteria: ≥ 18 years, UK-based, informed consent capable. Sub-study exclusions ensure safety (pacemakers, contact dermatitis), feasibility (travel restrictions), and cohort purity (no pain conditions).

Inclusion Criteria	Exclusion Criteria
Age over 18 years	Unable to attend or visit the research site *
	Inability to give full informed consent
	Self-reported pregnancy at the time of screening
Willing to complete pain diary and wearable sensor usage	Other neurological disease associated with gross structural or functional abnormalities of the central nervous system
	Any chronic pain condition
	Planned surgery within a month of screening
	Presence of pain or waiting for pain investigation
	Travel abroad in the plan duration of wearable usage
	Unable or unwilling to use body sensor for any reason (e.g., contact dermatitis over wear zone, implanted pacemaker or defibrillator, allergy or hypersensitivity to nickel)

* Participants are eligible for the study regardless of their location, as long as they are willing and able to travel to the research site.

### Participation procedure

**Fig 2:**
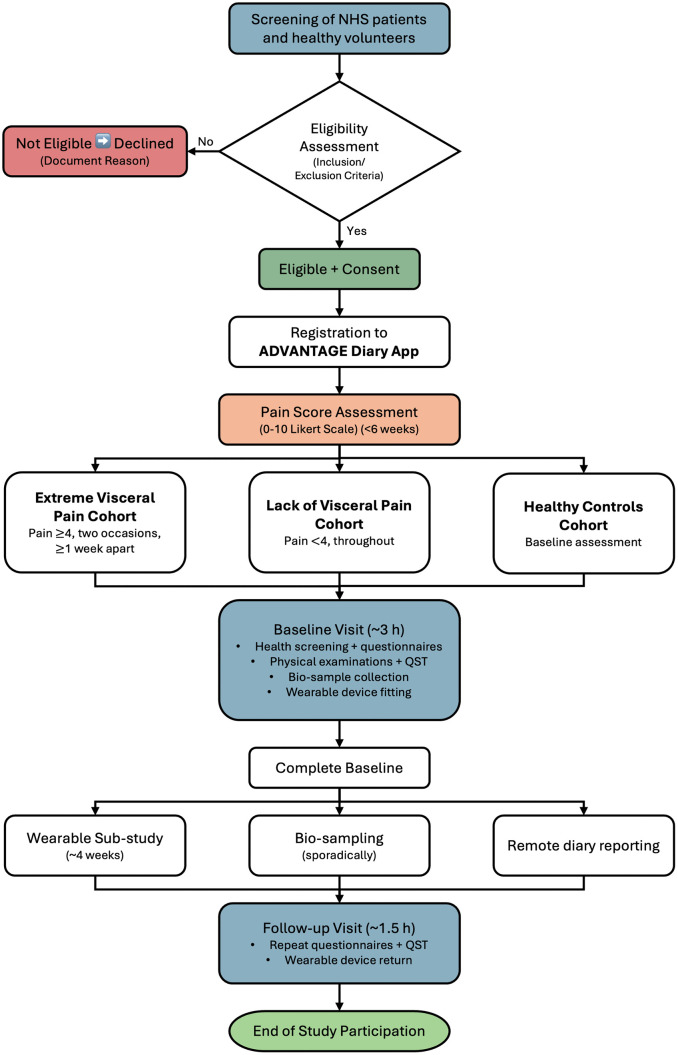
Participant Recruitment and Cohort Assignment Flow Diagram. Flowchart depicts screening, eligibility assessment, randomisation, and cohort assignment processes. NHS patient referrals and healthy volunteer recruitment pathways lead to initial screening using inclusion/exclusion criteria (Table 1). A six-week *ADVANTAGE Diary* remote mobile app phase determines cohort assignment: Extreme Visceral Pain (pain ≥4 on two occasions ≥1 week apart), Lack of Visceral Pain (pain <4 throughout), Healthy Controls (baseline only). Subsequent baseline/follow-up visits include optional wearable sub-study and bio-sampling as per eligibility. Decision points and participant progression shown chronologically.

The complete flow of participation is described in Fig 2, from recruitment to end of study. Participation in the study is entirely voluntary, and participants may withdraw at any time for any reason or be withdrawn by the Investigator. Data collected before withdrawal will be retained, identified only by Study ID, and will be fully anonymized. Choosing not to participate or withdrawing from the study will not affect the healthcare access or legal rights of participants. Participants attending onsite research visits will be reimbursed up to £15 per visit, in accordance with local site policies.

### Remote mobile app study

If the participant consents, they will be provided with a link to download the *ADVANTAGE Diary* remote mobile app. The app is hosted on a UK-based server that complies with General Data Protection Regulations (GDPR). Data access is protected using HTTPS and OpenID Connect 1.0 standards, with authentication managed through industry-standard OAuth 2.0 protocols. *ADVANTAGE Diary* is designed to meet Data Security Standard 4, equivalent to NHS hosting requirements. Participants will give informed e-consent via *ADVANTAGE Diary*, which will collect necessary information, such as their email address and a unique identifier generated by the app.

### On-site study visit

Informed consent will be obtained either electronically or on paper at the start of the on-site visit. Electronic or scanned paper consent forms will be securely stored in the University of Cambridge Clinical School Data Safe Haven. Each participant will be assigned a unique Study ID, which will also be securely stored. Explicit consent will be sought for (a) genetic analyses, (b) planned use of serum for *in vitro* or *in vivo* (mouse) studies, and (c) storage of serum and other samples (e.g., urine, vaginal swabs, faeces) for use in future ethically approved research. Individualized results from the analysis of these samples will not be disclosed to participants or their clinicians.

Only participants who have completed the mobile app sub-study are eligible for the wearable sub-study.

### Baseline on site visit assessments

#### Remote mobile app.

The *ADVANTAGE Diary* remote mobile app will be used to intermittently track pain experiences [20]. Participants will be asked to download the app from major platforms (e.g., App Store, Play Store) and will be registered by the study team with a unique study identifier. Over a period of up to 6 weeks, individuals will use the app to rate their visceral pain by responding to the prompt, “Please rate the severity of your pain right now,” on an 11-point Likert scale. The scale ranges from 0, representing no pain, to 10, representing the worst pain imaginable. This will specifically relate to their visceral pain. Individuals are encouraged to record their pain daily, and during painful “flares” as needed. The *ADVANTAGE Diary* remote mobile app will send reminders, no more than once daily, for these ratings. During this 6-week period, participants reporting a pain score of 4 or higher on two separate occasions at least one week apart will be placed in the Extreme Pain cohort, while those with scores below 4 will join the Lack of Visceral Pain cohort. This distinction is made to simplify cohort classification and study procedures. It does not imply that participants with scores below 4 are pain-free, nor that a score of 4 or 5 necessarily represents extreme pain; rather, it provides a practical framework for grouping participants based on pain intensity levels.

In exceptional cases where individuals face difficulties accessing the app due to IT literacy or lack of smartphone access, a telephone screening will be arranged. The study team will ask participants to rate their pain over the phone, using the same 0–10 scale.

### Structured clinical interview

Demographic information (age, race, sex at birth, self-identified gender) will be collected along with detailed medical history, including visceral disease diagnosis, family and menstrual history (for female at birth participants), history of surgeries, medications, and disease activity scores (see Supplementary Information for genetic sampling criteria).

The participants will also fill the following questionnaires:

1 Brief Pain Inventory-Short-Form [21].
2 Body maps to indicate locations of pain (see Supplementary Information for examples) [22,23].
3 McGill Pain Questionnaire [24].
4 O’Leary-Sant Interstitial Cystitis Questionnaire (IC-Q) [25].
5 Pelvic Pain Urgency/Frequency Patient Symptom scale [26].
6 ACR 1990 Fibromyalgia Diagnostic Criteria [27].
7 ACR 2016 Fibromyalgia Diagnostic Criteria [28].
8 Malmo POTS Score (MAPS) [29].

Standard measurements, including weight, height, pulse, and blood pressure, will be taken [30].

### Wearable sensor baseline

Prior to subsequent assessments, participants from Extreme Visceral Pain and Healthy Controls cohorts will be fitted with a Sibel Discovery *ANNE* chest sensor to establish a physiological baseline. The sensor will be applied at rest to collect continuous data including heart rate, skin temperature, and movement parameters. This baseline will serve as a reference point for future longitudinal comparisons and will contextualise results from later stages of the study, including physical examination, quantitative sensory testing, and bio-sampling.

### Quantitative sensory testing

Quantitative Sensory Testing (QST) will be performed to check for neuropathies [31,32] in all cohorts. Tests will include warm and cool detection with a contact heat thermode, vibration sense using a tuning fork, and touch sensitivity with von Frey filaments. Pressure pain thresholds will also be tested using an algometer. In cases where individuals lack expected pain sensitivity, a specialized protocol will be used to further assess painlessness (REC 12-EE-0369) [18].

### Bio-sampling

Participants from Extreme Visceral Pain and Lack of Visceral Pain cohorts will be asked to provide various samples to help researchers understand the similarities and differences in how conditions affect chronic visceral pain. Participants may choose to donate some samples while opting out of others, including those used in research involving non-human models.

**Whole blood:** Up to 50 mL of whole blood will be obtained through venepuncture. 5 mL of the whole blood sample will be sent for DNA extraction at the Department of Medical Genetics, University of Cambridge. 40 mL of the whole blood sample will be processed and sent for analysis and long-term storage to the Wolfson Sensory, Pain and Regeneration Centre at King’s College London. 5 mL of the whole blood sample will be processed and sent for long term storage to the Centre for Reproductive Health, Institute for Regeneration and Repair, University of Edinburgh.

**Urine:** Participants will collect mid-stream urine samples using a sterile device (UNI0054 or Colli-pee) at home or in the clinic, returning the samples via post. The urine sample will be sent to the Centre for Reproductive Health, Institute for Regeneration and Repair, University of Edinburgh.

**Vaginal swab:** Participants will self-collect a low vaginal swab (using BBLtm culture Swab MaxV or OMNIgene•vaginal | OMR-130), following provided instructions, and return the sample by post. The vaginal swab samples will be sent to the Centre for Reproductive Health, Institute for Regeneration and Repair, University of Edinburgh.

**Faeces:** Participants will collect up to three samples (using OMNIgene•GUT | OMR-200) at home and send them by post to the Cambridge Institute for Medical Research, University of Cambridge.

### Wearable sensors

**Fig 3:**
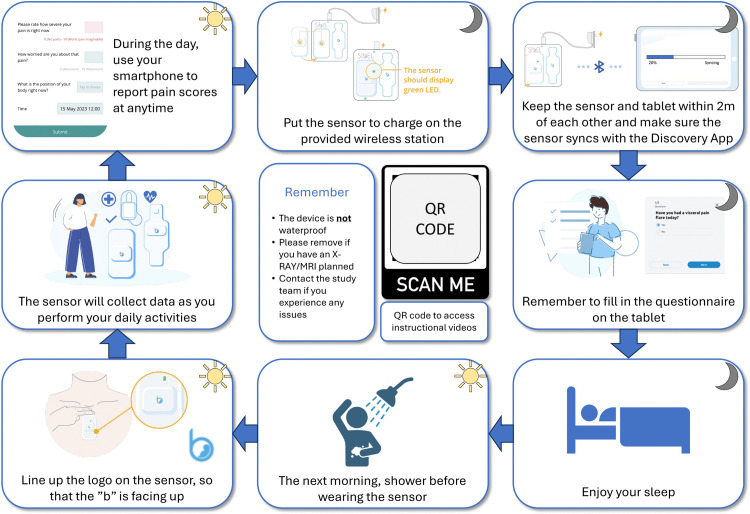
Wearable Sensor Deployment and Participant Instructions for the ADVANTAGE Study. Chest-worn respiratory sensor and wrist-worn physiological monitor setup for 4-week continuous data collection from Extreme Visceral Pain and Healthy Controls cohorts. Diagram illustrates wireless charging station, 2-metre Discovery App tablet sync range for data upload, daily reapplication protocol (shower before reattachment, correct orientation), and smartphone app for *ad libitum* pain reporting (0-10 Likert scale). Key operational constraints shown: non-waterproof (daily removal for showering), medical imaging contraindication (X-ray/MRI interference), QR code access to instructional videos. Ensures compliance for primary endpoint analysis correlating respiration rate, HRV, activity, and posture against real-time pain scores.

Participants from Extreme Visceral Pain and Healthy Controls cohorts will use two types of wearable sensors to monitor physiological data:

**Chest *wearable sensor*:** The Discovery *ANNE* Chest System is a flexible, rechargeable sensor worn on the upper chest for up to 16 hours daily over 30 days. It tracks heart rate, respiratory rate, skin temperature, and activity level. Participants will answer two questions daily via a mobile app in a tablet linked to the sensor: “Have you had a visceral pain flare today?” and “Have you changed or cancelled an activity due to visceral pain today?”. To encourage adherence to the wearable sensor usage protocol, instructional videos on how to wear the devices were made publicly available and shared with participants (see Supplementary Information). Additionally, Fig 3 is printed and shared with the participants to provide a visual guide with key steps for sensor setup, syncing, and daily use.

**Upper limb *smartwatch*:** The wrist-worn device (ActiGraph GT9X Link or GENEActiv, for Cambridge and Edinburgh sites, respectively) records limb movement and position continuously for up to 90 days. Data is downloaded after the wearable is returned, either by post or during a follow-up visit.

***ADVANTAGE Diary* remote mobile app questions during wearable sub-study:** Pain severity will continue to be recorded through the *ADVANTAGE Diary* app using the same 11-point scale as with the cohort classification stage. Participants will also receive daily reminders, and those who report pain will be asked additional questions: “How worried are you about that pain?” (rated 0–10), and “What is the position of your body right now?” (options include sitting, standing, lying down, squatting, moving actively). Those using the smartwatch and the *ADVANTAGE Diary* remote mobile app will be invited to continue for up to 90 days or until the smartwatch battery runs out after the follow up visit.

### Data management

Data will be collected using web-based database applications such as REDCap (Research Electronic Data Capture) and may also include paper-based Case Report Forms (CRFs). Where no prior record exists, the CRF will serve as the source document, and data will be directly entered into the CRF or REDCap. Clinical phenotype data will be extracted from hospital records by the clinician or research nurse and securely entered into the encrypted REDCap database. This database, developed by the ADVANTAGE research team, will be stored within the University of Cambridge’s safe haven.

Core data will include information from structured clinical interviews, physical examinations, questionnaires, and QST testing. Personal data (such as first/last names, email addresses, and NHS or CHI numbers) will also be collected, kept in the safe haven, and linked to each participant via a unique study ID generated by REDCap. This ID will be free of personal identifiers and consist of a 6-character string (e.g., LFWXAY). Access to the safe haven will be restricted to specific research team members authorized by the Chief Investigator. All other data (such as wearable sensor data) will be anonymized and stored outside the safe haven, linked only to the study ID.

Any fully anonymized material or data generated by the study (excluding personal data) may be transferred to external parties via a Data or Material Transfer Agreement. Identifiable information will be stored for up to one year after the study’s conclusion to ensure participant safety and to allow for regulatory review. Fully anonymized data (raw or processed) may be retained for up to five years beyond the study’s completion.

### Sample management plan

Any remaining samples from the study will be stored anonymously for use in future ethically approved research.

Serum samples will be linked-anonymized and stored at −70 to −90°C in secure, card-access-only freezers for the duration of the study. These samples will be sent to the Wolfson Sensory, Pain and Regeneration Centre at King’s College London for analysis and storage. Autoantibodies will be purified from the serum to investigate their autoreactivity, including whether they bind to tissues in mice and humans. Tests will be conducted to determine if serum or purified antibodies can replicate symptoms experienced by participant donors using behavioural tests in mice. The antibody-depleted serum will also be stored at −70°C and used for biochemical assays, such as measuring inflammatory markers. In addition, some serum samples will be sent to the Centre for Reproductive Health, Institute for Regeneration and Repair, University of Edinburgh for further research into chemicals and proteins associated with chronic pain.

Whole blood samples will be linked-anonymized and sent to the Department of Medical Genetics at the University of Cambridge, where DNA will be extracted and stored at −70 to −90°C in secure freezers. Using advanced or future-developed laboratory techniques, we will study changes in genomes that could influence the development or prevention of visceral pain. These linked-anonymized DNA samples will be fully anonymized upon study completion and may be stored indefinitely for future research.

Urine and vaginal swab samples will be linked-anonymized and sent to the Centre for Reproductive Health, Institute for Regeneration and Repair, University of Edinburgh. These samples will be stored in secure, card-access-only facilities and used for future ethically approved studies aimed at understanding chronic visceral pain. Specifically, we will study the role of hormones and bacteria in the body from these. Upon study completion, the linked-anonymized samples will be fully anonymized and may be stored indefinitely for future research.

Faecal samples will be linked-anonymized and sent to the Cambridge Institute for Medical Research at the University of Cambridge. They will then be batch-processed by a commercial collaborator for analysis. The analysis will help us better understand the role of gut bacteria in chronic visceral pain. These samples will be fully anonymized after the study and may be stored indefinitely for future research.

### Data analysis plan

The primary goal of this study is to investigate chronic visceral pain across different phenotypes using wearable sensor data and genetic analysis. While our broader objectives focus on generating exploratory insights into the genetic regulation of visceral pain, we aim to prioritize a well-powered, clinically useful outcome based on wearable sensor data, which will allow us to test a hypothesis within a smaller sample size.

### Primary endpoint analysis

The primary hypothesis we will test is that specific physiological parameters measured by wearable sensors (such as heart rate, physical activity, or respiration) will associate with self-reported pain intensity and specific body postures, such as sitting or lying down. We hypothesize that:

- Higher pain scores will associate with reduced mobility and specific postures (e.g., sitting or lying down).
- Wearable sensor data will provide objective measures that can predict or associate with self-reported pain intensity.

This hypothesis will be tested using a linear multiple regression model with the following four predictors:

1 Heart Rate Variability
2 Physical Activity (as measured by accelerometery)
3 Posture (sitting, standing, lying down)
4 Mean daily respiration rate

We expect that a sample size of n = 100 exclusively for the wearable sub-study provides 80% power to detect a moderate effect size (f² = 0.12 or larger) at p < 0.05, assuming four predictors.

For the primary analysis, we will fit a multiple linear regression model with mean daily visceral pain intensity as the dependent variable and the four pre-specified wearable predictors as independent variables. Model diagnostics will include inspection of residual-versus-fitted plots, Q–Q plots, and tests to assess both normality of residuals and multicollinearity between predictors. If substantial non-linearity or heteroscedasticity is detected, we will explore appropriate transformations of predictors or outcome while retaining the same pre-specified predictor set. All analyses will be conducted using validated statistical packages (either in R or Python), and the final model specification, diagnostics, and code will be documented to support reproducibility.

These four predictors were selected *a priori* based on established physiological relationships with pain states. Heart rate variability reflects autonomic nervous system engagement known to correlate with pain perception and flare severity in visceral pain syndromes [33–35]. Physical activity reduction is an established behavioural marker of pain avoidance and flare severity [36–38]. Posture is critical in visceral pain as many patients adopt characteristic positions during flares (e.g., lying down during endometriosis flares) [39,40]. Respiration rate was selected over alternative markers (such as skin temperature or sleep metrics) because: (i) it is directly modulated by nociceptive signalling through brainstem-vagal pathways critical to visceral pain processing [41,42], (ii) respiratory patterns are objectively measured with high fidelity by our chest-worn sensors, (iii) respiration is feasible for continuous long-term monitoring without imposing additional participant burden. Skin temperature was not selected due to confounding by environmental factors, and sleep metrics, whilst potentially informative, require longer data epochs incompatible with our primary real-time flare detection objective. No additional predictors will be included in this primary model.

We will use the correlation coefficients and regression analyses described to evaluate associations between these parameters and self-reported pain intensity, measured on an 11-point Likert scale (0–10). Confounding variables such as age, diagnosis, and occupation will not be included in the primary model but will be explored in sensitivity analyses.

Where multiple days of valid wearable data are available per participant, we will first derive participant-level mean values for each predictor over the observation period and use these aggregated values in the primary regression model; this avoids inflating the effective sample size and simplifies interpretation at the participant level. Day-level models will also be explored by using linear mixed-effects models with a random intercept for participant data.

Given that not all participants from the Extreme Pain cohort will use the wearables, the total number of participants we aim to recruit in the Extreme Pain cohort needs to exceed 100 to ensure adequate statistical power for this primary hypothesis. Healthy controls are not included in this confirmatory analysis, as they are unlikely to report sufficient pain scores for reliable modelling.

Missing data will be addressed using a complete-case analysis approach. No imputation methods will be applied for the wearable sensor data. Analyses will be restricted to participants with valid data for all variables included in the respective models. The extent and pattern of missingness will be reported descriptively. Where appropriate, we will perform sensitivity analyses to assess whether the exclusion of participants with missing data materially affects the results. For transparency, we will report the number of participants and observations included and excluded from each primary and sensitivity model in a CONSORT-style flow diagram and accompanying table. We will also present summary characteristics of participants with and without complete wearable data to allow readers to assess potential selection bias. This approach is justified by the observational nature of the study and the focus on real-world feasibility and data integrity.

This pre-specified analysis aligns with the SPIRIT recommendations for defining and justifying primary hypothesis-testing outcomes in clinical research protocols. All other objectives in this study, including genetic, immunological, and clustering analyses, are exploratory and will be pursued as hypothesis-generating efforts as more data becomes available.

### Key exploratory endpoint

The goal at the exploratory phase is to identify genetic variants that regulate visceral pain in people with visceral pain disorders, or in individuals who continue to experience pain despite remission or treatment. While we aim to conduct hypothesis-generating research, the genetic analysis will focus on identifying potential variants with moderate to strong effects, acknowledging that smaller effect sizes may require larger cohorts for reliable detection. The genetic approaches used have been proven effective in cohorts as small as 30–200 individuals with pain phenotypes [18]. However, we acknowledge that the requirement for ≥30 exomes per visceral disease pain phenotype may limit statistical power to detect genetic variants with modest effect sizes. Our analysis will explicitly report effect sizes, confidence intervals, and limitations related to sample size constraints in all genetic findings. Where modest effect sizes are detected, these will be flagged as requiring replication in larger cohorts. Findings will be reported in line with STREGA and STROBE guidelines for genetic association studies.

We note the following:

- At least 30 exomes are required per visceral disease pain phenotype to yield statistically valid results, in terms of the power to detect significant associations between genetic variants and pain phenotypes.
- DNA extraction failure rates are expected to be no more than 5% from blood samples and negligible from serum samples.

For this study, the following visceral pain phenotypes will be analysed, representing a broad spectrum of pain associated with visceral organ pathology:

1 Polycystic kidney disease
2 Inflammatory bowel disease
3 Post-vaginal mesh surgery
4 Chronic pancreatitis
5 Endometriosis
6 Painful bladder syndrome

Additionally, similar analyses will be conducted in the Lack of Visceral Pain cohort, where the severity of visceral disease does not correspond to expected levels of pain. This group will allow us to explore potential genetic mechanisms underlying discordant pain experiences. The following phenotypes will be included in this cohort:

1 Polycystic kidney disease
2 Inflammatory bowel disease
3 Chronic pancreatitis
4 Chronic pain syndromes (e.g., fibromyalgia, Complex Regional Pain Syndrome, migraine)
5 Cases of painlessness during percutaneous visceral organ procedures (e.g., biopsies)
6 Overactive bladder syndrome

This group will provide an exploratory dataset to investigate genetic variants that may regulate pain perception independent of disease severity.

**Mendelian gene effects** will be explored through multiple exome analyses, conducted in three steps:

1 Candidate genes
2 Genes identified in ADVANTAGE pre-clinical research as important for visceral pain in rodents
3 Agnostic analysis to discover unknown genes associated with visceral pain

These methods, widely used in UK Clinical and Medical Genetics practice, will compare results to local, national, and international normative databases.

**Functional SNP allele discovery (fSNPd)** will be used to detect Single Nucleotide Polymorphisms (SNPs) that may cause a pain phenotype triggered by environmental factors [43]. The approach is based on three principles:

- SNP alleles that cause fully penetrant effects are likely to be exonic and protein-changing (e.g., mis-sense, non-sense, indels, or splicing).
- Exome sequencing can detect most of these SNPs.
- Smaller cohorts are easier to collect, phenotype, and examine.

We will analyse exomes from multiple individuals to identify SNPs that occur at significantly different frequencies compared to the general population. Confounding variables will be minimized by matching subsets of control cohorts based on ethnicity or country. SNPs with significantly higher or lower allele frequencies in the study population will be identified through a two-tailed chi-squared test with Bonferroni and false discovery rate corrections applied.

Exome data will also be analysed for **mitochondrial genome variants** using methods developed in Professor Patrick Chinnery’s laboratory at the MRC Mitochondrial Biology Unit. Any findings are expected to be mosaic. These results will be compared to established normative ranges for human mitochondrial variants.

Mitochondrial variants will be evaluated for **heteroplasmy levels** and potential **mosaic/somatic variants** using established bioinformatics pipelines and clinical reporting thresholds appropriate for the specific analysis platform and cohort characteristics. Variant calling and heteroplasmy quantification will follow current best practices as defined by relevant clinical genomics standards and validated reference databases. All mitochondrial variant findings will be interpreted in the context of established population frequency data and clinical significance guidelines current at the time of analysis.

All exome and mitochondrial analyses will be performed using established, version-controlled bioinformatics pipelines, and we will document the exact software versions, parameter settings, and reference genomes used in a methods appendix or subsequent data descriptor.

### Secondary endpoint analyses

Descriptive statistics will be used to summarize the presence and concentration of **autoantibodies** in the cohorts. Regression models will explore potential associations between autoantibody levels and pain severity, adjusting for confounding variables such as age, gender, and disease type. Model assumptions for these regression analyses will be examined using the same diagnostic framework as for the primary endpoint, and, where appropriate, autoantibody concentrations will be transformed to better meet normality assumptions. This analysis may reveal immune-mediated mechanisms that contribute to persistent pain in certain individuals.

Clustering techniques will be applied to **phenotype data** to identify subgroups of participants who share similar clinical characteristics. These subgroups will then be analysed for differences in pain severity, pain location (using body maps), and wearable sensor data. Correlations between phenotypic clusters and genetic data will be assessed to explore potential genetic underpinnings of each phenotype.

Descriptive statistics will be provided for **body map-based** pain location frequencies [22,44], stratified by variables such as age group, diagnosis, gender, and pain interference scores. Hierarchical clustering will be used to discover novel pain phenotypes [45]. We will pre-specify the distance metric (Euclidean distance on scaled variables) and linkage method, and we will report the criteria used for selecting the final number of clusters, so that the clustering procedure can be replicated. Incomplete body map data will not be included in descriptive statistics, but the degree of missingness will be quantified and analysed for associations with other parameters, such as age and pain location.

### Study status

Recruitment for the study began on 1st November 2023 and is currently ongoing. Completion of participant recruitment is anticipated by 30th June 2026. Data collection is being conducted in parallel with recruitment and is expected to conclude by 31st December 2026. No data analysis has been performed, and no results have been generated at this stage. Preliminary results are expected to be available by 30th June 2027, following the completion of data collection and initial data processing. The current manuscript describes the study protocol only and does not include or imply any outcome data.

### Ethics and dissemination

This study involves human participants and was approved by the Health Research Authority, Health and Care Research Wales (23/PR/058), and the London-Surrey Research Ethics Committee. The Cambridge University Hospitals NHS Foundation Trust and the University of Cambridge are joint sponsors of the study (Sponsor No. A096518, IRAS ID 322886).

All participants provide written informed consent prior to enrolment in the study. Signed consent forms are securely stored in accordance with NHS and sponsor policies. No verbal consent procedures are used, and no consent waivers were granted by the approving ethics committees. Template consent forms for each participant cohort are provided in the Supplementary Information.

The study will follow the principles of the International Conference on Harmonization’s Guideline for Good Clinical Practice (ICH GCP). The Chief Investigator is responsible for the study’s overall conduct and compliance and may delegate certain responsibilities to appropriately trained staff.

Participation in the study is voluntary, and participants will be provided with Information Sheets (see Supporting Information). The Investigator must ensure all staff are informed about the study protocol and their specific duties. Local Investigator Site Files (ISFs) will contain all required documentation.

Researchers with data access must be trained in data protection regulations, including the UK General Data Protection Regulation and Data Protection Act 2018. All data will be securely stored, and clinical information will only be released with the participant’s written permission. Personal data will be held in a secure safe haven, accessible only via multi-factor authentication, and anonymized linked data will require usernames and passwords for access.

Ownership of the study data resides with the protocol lead and co-lead authors. Results will be disseminated through peer-reviewed publications, lay summaries, and collaborations with the Patient Charity Advisor Board. Published results will not contain personal data or any information that could lead to re-identification.

### Patient and public involvement

Patients were involved in the design, conduct, reporting, and dissemination plans of this research through the ADVANTAGE Patient and Charity Advisory Board. A summary of the Board’s contributions to the study and protocol can be found in the Supplementary Information.

## Supporting information

S1 Table Criteria for Genetic Sampling and Analysis. Detailed clinical and phenotypic criteria for exome sequencing sub-study selection across visceral pain cohorts (polycystic kidney disease, IBD, pancreatitis, endometriosis, bladder syndrome), including disease activity thresholds, family history requirements, and exclusion criteria for Mendelian analysis.
(DOCX)

S1 Appendix Patient and Public Involvement (PPI) Report. PCAB composition, meeting minutes, and contributions to diary app design, recruitment materials, and wearable sub-study feasibility assessment following NIHR PPI guidance standards.
(DOCX)

S2 Appendix SPIRIT 2013 Checklist. Completed 33-item SPIRIT checklist documenting protocol adherence to standard protocol items: recommendations for interventional trials (Chan et al., 2013, BMJ).
(DOCX)

S3 Appendix Participant Invitation Letter. NHS clinical team template for inviting eligible patients from visceral pain clinics to screening, with consent-for-contact permissions and study overview.
(PDF)

S4 Appendix Extreme Visceral Pain Cohort – Information Sheet and Consent Form. 17-page PIS/CF for participants with diary pain ≥4 (two occasions ≥1 week apart), detailing 4-week wearable monitoring, bio-sampling, and genetic analysis options.
(PDF)

S5 Appendix Lack of Visceral Pain Cohort – Information Sheet and Consent Form. 15-page PIS/CF for participants with diary pain <4 despite active visceral disease, covering baseline assessments and optional bio-sampling.
(PDF)

S6 Appendix Healthy Controls Cohort – Information Sheet and Consent Form. 12-page PIS/CF for age/gender-matched volunteers without chronic pain, including 4-week wearable monitoring and baseline QST assessments.
(PDF)

S1 Video Chest Sensor – Package Contents and Initial Setup. Demonstration of wireless charging station unboxing, sensor components inventory, tablet Discovery App pairing, and first-time activation.
(MP4)

S2 Video Chest Sensor Daily Application Protocol. Step-by-step morning reapplication post-shower, skin preparation, correct orientation, adhesion technique, and fit verification.
(MP4)

S3 Video Chest Sensor Removal, Cleaning, and Data Synchronization. End-of-day removal procedure, adhesive remover application, sensor cleaning protocol, and Discovery App data sync/upload.
(MP4)
