# Supplementary material for: ADVANTAGE: Advanced discovery of visceral analgesics by neuroimmune targets and the genetics of extreme human phenotype, a study protocol
Source: PLoS One. 2026 May 21;21(5):e0350169. doi: 10.1371/journal.pone.0350169 (PMC13193507; doi:10.1371/journal.pone.0350169)
Supplement: S1 Table — Detailed clinical and phenotypic criteria for exome sequencing sub-study selection across visceral pain cohorts (polycystic kidney disease, IBD, pancreatitis, endometriosis, bladder syndrome), including disease activity thresholds, family history requirements, and exclusion criteria for Mendelian analysis. (DOCX) [file pone.0350169.s001.docx]

# Criteria for Genetic Sampling and Analysis

| Autosomal Dominant Polycystic Kidney Disease | |
| --- | --- |
| Extreme visceral pain cohort | |
| Inclusion | - At least 2 pain ratings of 4 or higher, with at least one week between each rating - Genotyping or Radiological imaging according to standard criteria - Reported abdominal or loin pains of at least 3 months of duration |
| Exclusion | - Other types of PKD - An alternative explanation for chronic pain as judged by the referring or study team to be an exclusion (e.g. infected cysts, obstruction etc.) - Total bilateral nephrectomy - Any diagnostic work-up for investigation of pain - Surgical treatment planned for disease complication - Self-reported pregnancy or undergoing in-vitro fertilisation at the time of screening |
| Lack of expected pain cohort | |
| Inclusion | - Genotyping & Radiological imaging according to standard criteria - At least 2 pain ratings of 3 or lower, with at least one week between each rating - No reported pains that are perceived (or diagnosed) to be caused (or associated) with PKD in the past 3 months |
| Exclusion | - Total bilateral nephrectomy - 2 pain ratings of 4 or higher, with at least one week between each rating - Self-reported pregnancy or undergoing in-vitro fertilisation at the time of screening |

| Endometriosis | |
| --- | --- |
| Extreme visceral pain cohort | |
| Inclusion | - At least 2 pain ratings of 4 or higher, with at least one week between each rating - Assigned female at birth - Premenopausal - Diagnosis of endometriosis (based on imaging or surgical evidence) - Pain of at least 3 months localised to the true pelvis |
| Exclusion | - An alternative explanation for chronic pain as judged by the referring or study team to be an exclusion (e.g. infected cysts, obstruction etc.) - Women who are still being investigated for other causes of chronic pelvic pain - Self-reported pregnancy or undergoing in-vitro fertilisation at the time of screening - Surgery planned within the next six weeks |
| Lack of expected pain cohort | |
| Inclusion | - At least 2 pain ratings of 3 or lower, with at least one week between each rating - Assigned female at birth - Premenopausal - Endometriosis that is not painful |
| Exclusion | - Pain characteristic of, or associated with, endometriosis - 2 pain ratings of 4 or higher (*when they are not menstruating), with at least one week between each rating - Self-reported pregnancy or undergoing in-vitro fertilisation at the time of screening   Note: *dysmenorrhoea that does not require regular pain relief or impacts upon activities of daily living is not an exclusion criteria |

| Inflammatory bowel disease | | |
| --- | --- | --- |
| Ulcerative Colitis | | |
| Extreme visceral pain cohort | | |
| Inclusion | | - At least 2 pain ratings of 4 or higher, with at least one week between each rating - Established diagnosis of inactive ulcerative colitis ≥ 3 months, as judged by referring clinician - Unexplained abdominal pain or discomfort ≥ 3 months |
| Exclusion^1,2^ | | - Surgical treatment planned for disease complication - An alternative explanation for chronic pain as judged by the referring or study team to be an exclusion (e.g. active UC, infection, obstruction etc.) - Any diagnostic work-up for investigation of pain - Self-reported pregnancy or undergoing in-vitro fertilisation at the time of screening |
| Lack of expected pain cohort | | |
| Inclusion | | - At least 2 pain ratings of 3 or lower, with at least one week between each rating - Established diagnosis of currently active ulcerative colitis, as judged by referring clinician - No abdominal pain or discomfort for at least 3 months |
| Exclusion | | - 2 pain ratings of 4 or higher, with at least one week between each rating - Self-reported pregnancy or undergoing in-vitro fertilisation at the time of screening |
| Crohn’s disease | | |
| Extreme visceral pain cohort | | |
| Inclusion | - At least 2 pain ratings of 4 or higher, with at least one week between each rating - Established diagnosis of inactive Crohn’s disease ≥ 3 months , as judged by referring clinician - Unexplained abdominal pain or discomfort ≥ 3 months | |
| Exclusion | - An alternative explanation for chronic pain as judged by the referring or study team to be an exclusion (e.g. active Crohn’s, infected cysts, obstruction etc.) - Any diagnostic work-up for investigation of pain - Surgical treatment planned for disease complication - Self-reported pregnancy or undergoing in-vitro fertilisation at the time of screening | |
| Lack of expected pain cohort | | |
| Inclusion | - At least 2 pain ratings of 3 or lower, with at least one week between each rating - Established diagnosis of Crohn’s disease ≥3 months with active disease as judged by the referring clinician - Lack of reported pain during active disease | |
| Exclusion | - 2 pain ratings of 4 or higher, with at least one week between each rating. - Self-reported pregnancy or undergoing in-vitro fertilisation at the time of screening | |

| Chronic pancreatitis | |
| --- | --- |
| Extreme visceral pain cohort | |
| Inclusion | - At least 2 pain ratings of 4 or higher, with at least one week between each rating - Evidence of chronic pancreatitis as judged by the referring clinician |
| Exclusion | - Any feature of acute pancreatitis, that may account for pain (e.g. Abnormal lipase or amylase levels (at least more than three times the upper limit of normal based on local laboratory standard) features of systemic inflammatory response - Any diagnostic work-up for investigation of pain - An alternative explanation for chronic pain as judged by the referring or study team to be an exclusion (e.g. infected cysts, obstruction etc.) - Surgical treatment planned for disease complication - Self-reported pregnancy or undergoing in-vitro fertilisation at the time of screening |
| Lack of expected pain cohort | |
| Inclusion | - At least 2 pain ratings of 3 or lower, with at least one week between each rating, using the ADVANTAGE diary app - Chronic pancreatitis as judged by referring clinician (e.g. definite endoscopic or ultrasound evidence for, or proven and recurrent episodes of pancreatitis based on imaging or serological markers) |
| Exclusion | - Chronic pancreatitis requiring use of significant pain relief - 2 pain ratings of 4 or higher, with at least one week between each rating - Self-reported pregnancy or undergoing in-vitro fertilisation at the time of screening |

| Vaginal mesh insertion | |
| --- | --- |
| Extreme visceral pain cohort | |
| Inclusion | - At least 2 pain ratings of 4 or higher, with at least one week between each rating - Previous surgical mesh insertion for stress urinary incontinence or pelvic organ prolapse - Post-surgical pain for at least 3 months duration |
| Exclusion | - An alternative explanation for chronic pain as judged by the referring or study team to be an exclusion (e.g. infected cysts, ulcers etc.) - Surgical treatment planned for disease complication - Any diagnostic work-up for investigation of pain - Self-reported pregnancy or undergoing in-vitro fertilisation at the time of screening |

| Painful bladder syndrome | |
| --- | --- |
| Extreme visceral pain cohort | |
| Inclusion | - At least 2 pain ratings of 4 or higher, with at least one week between each rating - Pelvic pain, pressure or discomfort perceived to be related to the urinary bladder accompanied by urinary symptom(s) such as persistent urge to void or frequency - Symptoms for at least 3 months in absence of culture-positive persistent urinary tract infection or other obvious pathology or identifiable causes |
| Exclusion | - An alternative explanation for chronic pain as judged by the referring or study team to be an exclusion (e.g. foreign body, diverticulum, stones etc.) - Any diagnostic work-up for investigation of pain - Planned invasive procedures of the urinary tract - Self-reported pregnancy or undergoing in-vitro fertilisation at the time of screening |

| Percutaneous biopsy of visceral organs | |
| --- | --- |
| Lack of expected pain cohort | |
| Inclusion | - At least 2 pain ratings of 3 or lower, with at least one week between each rating - Repeated percutaneous procedures (e.g. pleural biopsies) of thoracic, abdominal or pelvic organs that routinely required analgesics. - Lack of (self-reported) pain associated with the procedure during or 1 week after the procedure - Able to undergo procedure without systemic or local analgesia |
| Exclusion | - Behaviour interpretated as pain by the proceduralist - 2 pain ratings of 4 or higher, with at least one week between each rating - Request for analgesia (systemic or local analgesia) for the procedure - Self-reported pregnancy or undergoing in-vitro fertilisation at the time of screening |

| Fibromyalgia | |
| --- | --- |
| Lack of expected visceral pain cohort | |
| Inclusion | - Meets the 2016 ACR criteria for Fibromyalgia - Has no visceral pain condition - At least 2 pain ratings of 3 or lower, with at least one week between each rating |
| Exclusion | - Self-reported pregnancy or undergoing in-vitro fertilisation at the time of screening - 2 pain ratings of 4 or higher, with at least one week between each rating |
