# Supplementary material for: ADVANTAGE: Advanced discovery of visceral analgesics by neuroimmune targets and the genetics of extreme human phenotype, a study protocol
Source: PLoS One. 2026 May 21;21(5):e0350169. doi: 10.1371/journal.pone.0350169 (PMC13193507; doi:10.1371/journal.pone.0350169)
Supplement: S1 Appendix — PCAB composition, meeting minutes, and contributions to diary app design, recruitment materials, and wearable sub-study feasibility assessment following NIHR PPI guidance standards. (DOCX) [file pone.0350169.s002.docx]

# **Patient and Public Involvement**

Early in the grant-writing process for the UKRI Advanced Pain Discovery Platform (APDP) Consortia, we sought to form a Patient and Charities Advisory Board (PCAB). We initially approached Tess Harris, who not only had a visceral disorder associated with pain but was also the CEO of the Polycystic Kidney Disease Charity (PKDC). This charity had previously been involved with research, was well-run, and offered multifaceted support to its members in the UK with Polycystic Kidney Disease.

Tess Harris agreed to head our PCAB, with Professor Amanda Williams as the Principal Investigator (PI) for ADVANTAGE research ethics. Over the next six months, Tess Harris recruited additional members for the PCAB and commenced bi-monthly meetings.

The PCAB was involved in grant editing at multiple stages. Tess Harris was one of the six members of the ADVANTAGE group interviewed by the MRC for the consortium award; she also contributed to the presentation during the interview. Since the APDP award, the PCAB has played a significant role in all clinical matters. Furthermore, PIs of ADVANTAGE presented to the PCAB either upon request or when clinical issues arose that required solutions. The PCAB also oversees our recruitment by disorder and by sex for both the main project and the Pain App.

The PCAB helped address or resolve the following issues:

- **Response to Interview Panel Questions**: Prepared answers prior to the interview.
- **Approach to Visceral Pain Groups**: Developed strategies for engaging with groups such as vaginal mesh and painful bladder syndrome patients.
- **Working Definitions**: Established definitions for severe pain, mild pain, and pain spasms.
- **Lay Summary for APDP Website**: Created an accessible summary of ADVANTAGE.
- **Website Design and Content**: Contributed to the design, logo, and content of the ADVANTAGE website.
- **Wearable Practicalities**: Addressed issues related to sending, applying, downloading data from, and returning wearables.
- **Instructional Videos Review**: Reviewed the videos made by the clinical team to instruct participants on using the wearables.
- **Publication and Authorship Guidance**: Provided guidelines for publications under ADVANTAGE.
- **PKD Pain Studies Collaboration**: Discussed the relationship and interaction between PKD pain studies and ADVANTAGE.
- **Pain Matters Article**: Assisted in creating an article about ADVANTAGE.
- **Recruitment Plans**: Developed initial plans for participant recruitment.
- **Participant Feedback**: Established mechanisms for collecting and addressing feedback from participants.
- **Travel Funds**: Organized travel funding for participants.
- **Pain Criteria Revisions**: Revised pain criteria for all eight visceral disorders after recruitment began.
- **Research Ethics Amendments**: Involved in all four major amendments to ADVANTAGE research ethics.
- **PCAB Expenses**.
- **Pharmaceutical Collaborations**: Facilitated collaborations with AstraZeneca, Holobiome, Eli Lilly, and other APDP studies.
