# Supplementary material for: ADVANTAGE: Advanced discovery of visceral analgesics by neuroimmune targets and the genetics of extreme human phenotype, a study protocol
Source: PLoS One. 2026 May 21;21(5):e0350169. doi: 10.1371/journal.pone.0350169 (PMC13193507; doi:10.1371/journal.pone.0350169)
Supplement: S2 Appendix — Completed 33-item SPIRIT checklist documenting protocol adherence to standard protocol items: recommendations for interventional trials (Chan et al., 2013, BMJ). (DOCX) [file pone.0350169.s003.docx]

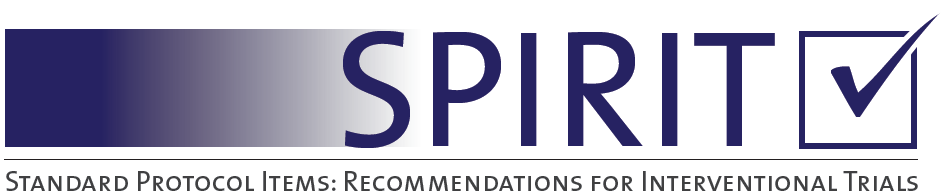


SPIRIT 2013 Checklist: Recommended items to address in a clinical trial protocol and related documents*

| Section/item | ItemNo | Description | Position in the manuscript |
| --- | --- | --- | --- |
| **Administrative information** | | | |
| Title | 1 | Descriptive title identifying the study design, population, interventions, and, if applicable, trial acronym | Detailed in the **Title page** of the manuscript: *"Clinical ADVANTAGE: Advanced Discovery of Visceral Analgesics by Neuroimmune Targets and the Genetics of Extreme Human Phenotype"* |
| Trial registration | 2a | Trial identifier and registry name. If not yet registered, name of intended registry | Section **Ethics, Registration and Dissemination**. Cambridge University Hospitals NHS Foundation Trust. Sponsor No. A096518, IRAS ID 322886 |
|  | 2b | All items from the World Health Organization Trial Registration Data Set | **Public title:** Advanced Discovery of Visceral Analgesics Study  **Scientific title:** Clinical ADVANTAGE: Advanced Discovery of Visceral Analgesics by Neuroimmune Targets and Genetics of Extreme Human Phenotype  **Intervention:** This study is observational; no interventions are involved.  **Primary outcome:** Identification of functional pathways involved in chronic visceral pain.  **Secondary outcomes:** Pain mapping, wearable sensor analysis, bio-sample findings.  **Study type:** Observational cohort study.  **Date of registration:** (date to be added upon registration).  **Target sample size:** 700 participants.  **Recruitment status:** Recruiting.  **Sponsors:** University of Cambridge and Cambridge University Hospitals NHS Foundation Trust |
| Protocol version | 3 | Date and version identifier | IRAS 322886 Clinical ADVANTAGE study protocol V1.5_19Jun24 |
| Funding | 4 | Sources and types of financial, material, and other support | Described in the **Funding** section of the manuscript |
| Roles and responsibilities | 5a | Names, affiliations, and roles of protocol contributors | Contributor roles are detailed on the **Title page** and the **Footnotes** section (e.g., **Marco Vinicio Alban-Paccha** as the lead author) |
|  | 5b | Name and contact information for the trial sponsor | Included in the **Footnotes** section, where the trial joint sponsors: University of Cambridge and Cambridge University Hospitals NHS Foundation Trust, are listed. |
|  | 5c | Role of study sponsor and funders, if any, in study design; collection, management, analysis, and interpretation of data; writing of the report; and the decision to submit the report for publication, including whether they will have ultimate authority over any of these activities | Addressed under **Funding** and **Contributors** section |
|  | 5d | Composition, roles, and responsibilities of the coordinating centre, steering committee, endpoint adjudication committee, data management team, and other individuals or groups overseeing the trial, if applicable (see Item 21a for data monitoring committee) | Addressed under **Funding** and **Contributors** section |
| **Introduction** | | | |
| Background and rationale | 6a | Description of research question and justification for undertaking the trial, including summary of relevant studies (published and unpublished) examining benefits and harms for each intervention | Thoroughly explained in the **Introduction** section. This covers the knowledge gap in visceral pain management and the need for research |
|  | 6b | Explanation for choice of comparators | **Participant Recruitment**. Healthy Volunteers used as matched controls |
| Objectives | 7 | Specific objectives or hypotheses | Clear objectives are outlined in the **Aims and Endpoints** section, including primary and secondary goals of understanding visceral pain mechanisms. |
| Trial design | 8 | Description of trial design including type of trial (eg, parallel group, crossover, factorial, single group), allocation ratio, and framework (eg, superiority, equivalence, noninferiority, exploratory) | Described in the **Methods** and **Study Design** sections, specifically highlighting that this is an observational cohort study with three groups. |
| Methods: Participants, interventions, and outcomes | | | |
| Study setting | 9 | Description of study settings (eg, community clinic, academic hospital) and list of countries where data will be collected. Reference to where list of study sites can be obtained | The study setting is described as taking place in clinical settings, with participants recruited from the UK. Section **Participant Recruitment** |
| Eligibility criteria | 10 | Inclusion and exclusion criteria for participants. If applicable, eligibility criteria for study centres and individuals who will perform the interventions (eg, surgeons, psychotherapists) | Clearly outlined in the **Methods** section, with specific inclusion and exclusion criteria for each cohort. |
| Interventions | 11a | Interventions for each group with sufficient detail to allow replication, including how and when they will be administered | Not applicable as this is an observational study, so no interventions are assigned. |
|  | 11b | Criteria for discontinuing or modifying allocated interventions for a given trial participant (eg, drug dose change in response to harms, participant request, or improving/worsening disease) | Not applicable (no interventions in this observational study). |
|  | 11c | Strategies to improve adherence to intervention protocols, and any procedures for monitoring adherence (eg, drug tablet return, laboratory tests) | Not applicable as this study does not involve interventions. |
|  | 11d | Relevant concomitant care and interventions that are permitted or prohibited during the trial | Not applicable as there are no interventions or related care protocols for this observational study. |
| Outcomes | 12 | Primary, secondary, and other outcomes, including the specific measurement variable (eg, systolic blood pressure), analysis metric (eg, change from baseline, final value, time to event), method of aggregation (eg, median, proportion), and time point for each outcome. Explanation of the clinical relevance of chosen efficacy and harm outcomes is strongly recommended | Clearly defined in the **Aims and Endpoints** section. |
| Participant timeline | 13 | Time schedule of enrolment, interventions (including any run-ins and washouts), assessments, and visits for participants. A schematic diagram is highly recommended (see Figure) | The timeline for participant involvement is described in the **Study Design** and **Wearable Sub-study** sections, with clear steps for app use, on-site visits, and wearable data collection explained in **Figure 1**. |
| Sample size | 14 | Estimated number of participants needed to achieve study objectives and how it was determined, including clinical and statistical assumptions supporting any sample size calculations | The estimated sample size of 700 participants is mentioned in the **Recruitment** section, with details on how participants will be recruited. |
| Recruitment | 15 | Strategies for achieving adequate participant enrolment to reach target sample size | Recruitment strategies, including social media advertising and clinical referrals, are outlined in the **Recruitment** section. |
| **Methods: Assignment of interventions (for controlled trials)** | | | |
| Allocation: |  |  |  |
| Sequence generation | 16a | Method of generating the allocation sequence (eg, computer-generated random numbers), and list of any factors for stratification. To reduce predictability of a random sequence, details of any planned restriction (eg, blocking) should be provided in a separate document that is unavailable to those who enrol participants or assign interventions | Not applicable (no randomization or interventions in this observational study). |
| Allocation concealment mechanism | 16b | Mechanism of implementing the allocation sequence (eg, central telephone; sequentially numbered, opaque, sealed envelopes), describing any steps to conceal the sequence until interventions are assigned | Not applicable (no randomization or interventions). |
| Implementation | 16c | Who will generate the allocation sequence, who will enrol participants, and who will assign participants to interventions | Not applicable (no randomization or interventions). |
| Blinding (masking) | 17a | Who will be blinded after assignment to interventions (eg, trial participants, care providers, outcome assessors, data analysts), and how | Not applicable (no randomization or interventions). |
|  | 17b | If blinded, circumstances under which unblinding is permissible, and procedure for revealing a participant’s allocated intervention during the trial | Not applicable (no randomization or interventions). |
| **Methods: Data collection, management, and analysis** | | | |
| Data collection methods | 18a | Plans for assessment and collection of outcome, baseline, and other trial data, including any related processes to promote data quality (eg, duplicate measurements, training of assessors) and a description of study instruments (eg, questionnaires, laboratory tests) along with their reliability and validity, if known. Reference to where data collection forms can be found, if not in the protocol | Described in the **Data Collection** section. Data is collected via mobile app, on-site visits, and wearable sensors, with plans for bio-sample collection. |
|  | 18b | Plans to promote participant retention and complete follow-up, including list of any outcome data to be collected for participants who discontinue or deviate from intervention protocols | Addressed in the **Data Collection** section. |
| Data management | 19 | Plans for data entry, coding, security, and storage, including any related processes to promote data quality (eg, double data entry; range checks for data values). Reference to where details of data management procedures can be found, if not in the protocol | Data management plans, including anonymization, storage, and handling of personal information, are detailed in the **Data Management** section. |
| Statistical methods | 20a | Statistical methods for primary/secondary outcomes | Statistical methods for analysing primary and secondary outcomes are outlined in the **Analysis Plan** section. Confirmatory vs exploratory analyses clearly described |
|  | 20b | Methods for any additional analyses (eg, subgroup and adjusted analyses) | Subgroup analyses, including clustering of time-series data, are mentioned in the **Analysis Plan** section. |
|  | 20c | Definition of analysis population relating to protocol non-adherence (eg, as randomised analysis), and any statistical methods to handle missing data (eg, multiple imputation) | **Analysis Plan.** Missing data will be handled by multiple imputation in the case of pain ratings. |
| **Methods: Monitoring** | | | |
| Data monitoring | 21a | Composition of data monitoring committee (DMC); summary of its role and reporting structure; statement of whether it is independent from the sponsor and competing interests; and reference to where further details about its charter can be found, if not in the protocol. Alternatively, an explanation of why a DMC is not needed | The Data Managers are detailed in the **Data Management** and **Contributors** section. |
|  | 21b | Description of any interim analyses and stopping guidelines, including who will have access to these interim results and make the final decision to terminate the trial | Not applicable (observational study, no stopping rules). |
| Harms | 22 | Plans for collecting, assessing, reporting, and managing solicited and spontaneously reported adverse events and other unintended effects of trial interventions or trial conduct | Any adverse events require the stop of use of wearables. |
| Auditing | 23 | Frequency and procedures for auditing trial conduct, if any, and whether the process will be independent from investigators and the sponsor | Included in the **Footnotes** section, where the trial joint sponsors: University of Cambridge and Cambridge University Hospitals NHS Foundation Trust, will be in charge of auditing, if necessary. |
| Ethics and dissemination | | | |
| Research ethics approval | 24 | Plans for seeking research ethics committee/institutional review board (REC/IRB) approval | Mentioned in the **Ethics Registration and Dissemination** section. The study has been approved by the Cambridge University Hospitals NHS Foundation Trust. |
| Protocol amendments | 25 | Plans for communicating important protocol modifications (eg, changes to eligibility criteria, outcomes, analyses) to relevant parties (eg, investigators, REC/IRBs, trial participants, trial registries, journals, regulators) | Mentioned in the **Ethics Registration and Dissemination** section. The amendments will be approved by the Cambridge University Hospitals NHS Foundation Trust. |
| Consent or assent | 26a | Who will obtain informed consent or assent from potential trial participants or authorised surrogates, and how (see Item 32) | Consent procedures are described in the **On-Site Study** section. Participants provide informed consent either electronically or on paper during on-site visits. |
|  | 26b | Additional consent provisions for collection and use of participant data and biological specimens in ancillary studies, if applicable | Additional consent for bio-sample collection and use in future studies is included in the **On-Site Study** section. |
| Confidentiality | 27 | How personal information about potential and enrolled participants will be collected, shared, and maintained in order to protect confidentiality before, during, and after the trial | Confidentiality is addressed in the **Data Management** section, with personal information securely stored and anonymized. |
| Declaration of interests | 28 | Financial and other competing interests for principal investigators for the overall trial and each study site | Detailed in the **Footnotes**. No conflicts declared by the authors. |
| Access to data | 29 | Statement of who will have access to the final trial dataset, and disclosure of contractual agreements that limit such access for investigators | The dataset will be openly available 2 years after recruitment finishes. |
| Ancillary and post-trial care | 30 | Provisions, if any, for ancillary and post-trial care, and for compensation to those who suffer harm from trial participation | Participants in the study will not receive specific post-trial care, as the study is observational and does not involve experimental treatments. However, should any medical concerns arise during the course of the study, participants will be advised to seek appropriate medical care through their regular healthcare providers. |
| Dissemination policy | 31a | Plans for investigators and sponsor to communicate trial results to participants, healthcare professionals, the public, and other relevant groups (eg, via publication, reporting in results databases, or other data sharing arrangements), including any publication restrictions | Dissemination plans are outlined in the **Ethics Registration and Dissemination** section. The study plans to share results through public databases and publications. |
|  | 31b | Authorship eligibility guidelines and any intended use of professional writers | Authorship for any publications arising from this study will be determined based on the International Committee of Medical Journal Editors (ICMJE) criteria, which require substantial contributions to the conception or design of the work, or the acquisition, analysis, or interpretation of data; drafting or revising the manuscript critically for important intellectual content; and final approval of the version to be published. |
|  | 31c | Plans, if any, for granting public access to the full protocol, participant-level dataset, and statistical code | Plans for public data sharing are mentioned in the **Data Management** section. |
| Appendices | | | |
| Informed consent materials | 32 | Model consent form and other related documentation given to participants and authorised surrogates | Detailed in the Supplemental information |
| Biological specimens | 33 | Plans for collection, laboratory evaluation, and storage of biological specimens for genetic or molecular analysis in the current trial and for future use in ancillary studies, if applicable | Detailed in the **Bio-Sampling** section, which covers the collection, storage, and future use of biological specimens. |

*It is strongly recommended that this checklist be read in conjunction with the SPIRIT 2013 Explanation & Elaboration for important clarification on the items. Amendments to the protocol should be tracked and dated. The SPIRIT checklist is copyrighted by the SPIRIT Group under the Creative Commons “[Attribution-NonCommercial-NoDerivs 3.0 Unported](http://www.creativecommons.org/licenses/by-nc-nd/3.0/)” license.
