# Supplementary material for: ADVANTAGE: Advanced discovery of visceral analgesics by neuroimmune targets and the genetics of extreme human phenotype, a study protocol
Source: PLoS One. 2026 May 21;21(5):e0350169. doi: 10.1371/journal.pone.0350169 (PMC13193507; doi:10.1371/journal.pone.0350169)
Supplement: S3 Appendix — NHS clinical team template for inviting eligible patients from visceral pain clinics to screening, with consent-for-contact permissions and study overview. (PDF) [file pone.0350169.s004.pdf]

ADVANTAGE VISCERAL PAIN CONSORTIUM

UNIVERSITY OF CAMBRIDGE

NHS Cambridge University Hospitals NHS Foundation Trust

Invitee [name]
[Address]
[Address]
[Address]
POSTCODE

Department
Address
Address
Postcode

[dd-mm-yyyy]

Title of Project: Clinical ADVANTAGE Study

Name of Researcher: PI's name

Dear Sir/Ms,

We are writing to you as you have been identified by your clinical care team as a potential eligible for a research study.

We would like to invite you to participate in this research study that is trying to gain a better understanding of what it's like to have severe 'visceral' pain due to painful bladder syndrome, vaginal mesh complication, fibromyalgia, endometriosis, inflammatory bowel disease, autosomal dominant polycystic kidney disease and/or chronic pancreatitis.

We are looking to invite people to this study particularly those who have severe pain despite few signs of disease. We will also invite those who feel little or no pain despite disease.

By taking part in the study, you will have the opportunity to:

- Complete a daily mobile 'pain' app
- Attend up to 2 onsite visits (up to 3 hours) at Addenbrooke's Hospital Cambridge/ or Royal Edinburgh Infirmary
- Use 'wearable' sensors for up to 4 weeks whilst completing the app.

If you would like to find out more about the study or would like to take part, please get in touch with the research team either by email, or phone.

Email: study email

Phone: study team phone number

With best wishes,

Patient's consultant

scan for link to send email
