# Supplementary material for: ADVANTAGE: Advanced discovery of visceral analgesics by neuroimmune targets and the genetics of extreme human phenotype, a study protocol
Source: PLoS One. 2026 May 21;21(5):e0350169. doi: 10.1371/journal.pone.0350169 (PMC13193507; doi:10.1371/journal.pone.0350169)
Supplement: S6 Appendix — 12-page PIS/CF for age/gender-matched volunteers without chronic pain, including 4-week wearable monitoring and baseline QST assessments. (PDF) [file pone.0350169.s007.pdf]

Participant Information Sheet: Healthy volunteers

Title of Project:	Clinical ADVANTAGE Study
Name of Researcher:	Professor Geoff Woods

We invite you to take part in a research study.

- Before you decide to take part, it is important for you to understand why the research is being done and what it will involve.
- Please take time to read the following information carefully. Discuss it with friends and relatives if you wish.
    1. You are free to decide whether or not to take part in this study. If you choose not to take part, this will not affect the care you get from your own doctors.
- Ask us if there is anything that is not clear or if you would like more information.

Section 1 tells you the purpose of the study and what will happen if you take part

Section 2 gives you more detailed information about the conduct of the study

---

Section 1: Purpose of the study and what will happen

1. Why are we doing this study?

We still have a lot to understand about what it's like to have severe chronic 'visceral' pain (pain that comes from the inside of our bodies) in people with painful bladder syndrome, vaginal mesh complication, endometriosis, inflammatory bowel disease, autosomal dominant polycystic kidney disease, fibromyalgia, and chronic pancreatitis.

This information sheet describes the study for those who fulfill the criteria and have been identified as a healthy volunteer. You would be asked to attend two face-to-face visits at Addenbrooke's Hospital, Cambridge.

Currently, treatment for the pain from most visceral conditions is specific to the disease, as there are no analgesics targeting visceral pain. There has been little attention given to understanding the similarities and differences in people's experience of visceral pain. We want to understand why people with the same disease often have different pain experiences and why pain can persist even when the underlying condition is treated. We hope to discover the genetic and microbiome (the bacteria that live in our bodies) factors behind chronic visceral pain. We will also look to see if the immune system is involved, and how pain is experienced both physically (detected by the nervous system) and mentally (psychologically).

By studying the pain experiences of individuals with different visceral diseases, we hope to identify common factors as well as disease or organ-specific causes of visceral pain. This knowledge will help us develop new and more effective treatments for pain relief. Additionally, we aim to produce the tools to allow precision medicine, where treatments are tailored to meet the specific needs of individuals with chronic visceral pain.

2. Why am I being asked to take part?

You have been identified as a healthy volunteers aged over 18 years and you do not have any of the following conditions: endometriosis, inflammatory bowel disease, chronic pancreatitis,

autosomal dominant polycystic kidney disease, or have had biopsy of a major organ (such as lung, bowel or bladder). You are experiencing little or no visceral pain.

3. What would taking part involve?

If you would like to take part, we will ask you a few questions before you attend to make sure you are eligible based on our study criteria.

You will be asked to download and consent to using an App so that you can report your Pain Score to us prior to being invited to the research study. Before you start the ADVANTAGE diary app, the research team will set you up with access to the ADVANTAGE diary app. They will ask you to read the patient information leaflet and complete the electronic consent form. They'll also need some information about you, including name, and contact details (i.e., email address) which they will store securely. This score should reflect the level of pain that you feel in your abdomen and / or pelvis at the time that you input. This will allow us to ensure that we only recruit healthy volunteers who have little or no pain. In the exceptional circumstances that you are unable to use this App, the research team can ring you on two separate occasions more than 1 week apart to collect your Pain Score.

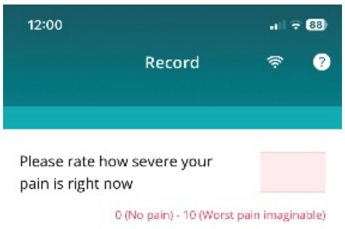

When you attend the face-to-face visit, the research team will discuss the study with you in more detail and confirm your eligibility, whilst giving you the opportunity to ask any questions you may have. If you agree to take part in the study, they will ask you to sign a consent form (electronic or paper), to confirm this.

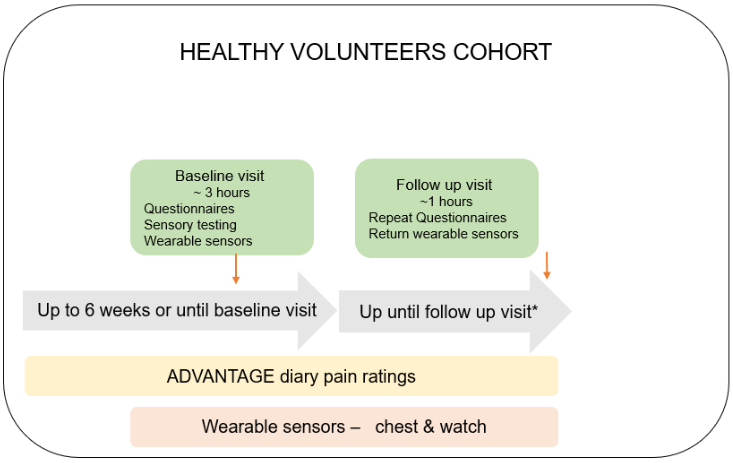

* Up to 4 weeks

The first onsite visit will last up to 3 hours during which the research team will ask you a few questions including ones about your medical history. They may also measure your weight, height and blood pressure and you will be asked to complete questionnaires about your pain experiences. The follow up visit will last up to one hour during which you will be asked to return the study sensors and repeat some of the questionnaires.

During your visits, the research team will complete 'sensory testing' on your hands and feet. This is to measure when you detect temperature or pressure changes, and when those changes start to feel uncomfortable, in other words, your pain threshold. To do this we will use

a variety of stimuli on your hands and feet. These include (i) hair filaments, (ii) vibrating 'tuning fork', (iii) using a temperature-controlled probe (iv) blunt rubber tip device.

We will also be measuring your tolerance for pressure and cold pain using calibrated devices. For pressure pain tolerance, a blood pressure cuff would be placed over your arm and slowly inflated until it becomes uncomfortable. For cold pain tolerance, you would be asked to place your hand in a temperature-controlled water bath for as long as you can. They will monitor your blood pressure and heart during this test.

You will be asked to rate the level of pain you experience during the tests. All tests are performed with calibrated devices with safety limits, and you can stop them at any time if you find them too uncomfortable.

We would also like you to use the wearable sensors that have been selected for this study – a chest skin sensor and a watch sensor.

The Discovery ANNE Chest System (or chest skin sensor) is a soft, flexible, skin-mountable biosensor that measures vital signs such as heart rate, respiratory rate, skin temperature and movement. The chest skin sensor is worn just below the suprasternal notch, or the space between where your collarbones meet.

While the Discovery ANNE Chest System is not currently CE marked, it has been approved by the U.S. Food and Drug Administration (FDA), and has been safely used in numerous studies, including those involving newborns and pregnant women.

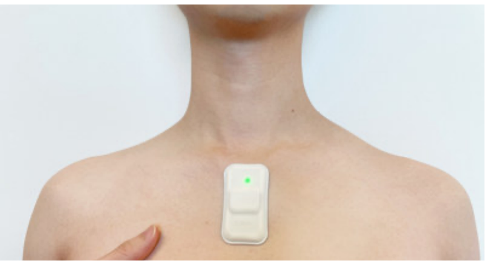

During your visit they will set up and let you know how to use the sensors and the electronic tablet it comes with. They will provide you with instructions on how to use the wearable sensors, including applying the adhesives, and provide video instructions for you to access at any time. They will ask you to wear the chest skin sensor and watch until the follow up visit (1-4 weeks after baseline visit), using a new adhesive for the chest skin sensor every morning, whilst taking it off at night to charge and download the data to the accompanying tablet.

There will be two questions within the Sibel app that ask about your pain over the day:

1. Have you had a visceral pain flare today?
2. Have you changed or cancelled an activity due to visceral pain today?

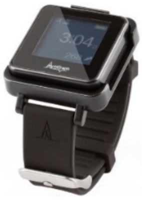

They'd also like you to wear a watch sensor that measures your movement and body position – like one of those in the pictures. The watch sensor does not have to be recharged and you can wear it all the time or until the battery runs out.

In the event of loss or damage, the study team would be liable to fund any replacements. This means that if the devices are lost, stolen, or damaged while in your possession, the study team will have to pay for them out of their own budget.

We kindly request that you take good care of the devices. This is very important because we do not have insurance coverage for the devices, and we have limited funds to replace them. We appreciate your cooperation and understanding.

The study team will book you in for a second visit so that you can return your sensors and ask you some questions related to your experience.

4. Do I have to take part?

It is completely up to you whether or not you would like to take part in the study.

If you decide not to, your decision will not affect the healthcare you receive in any way. If you do decide to join, you will be free to withdraw at any time and without having to give a reason.

5. What Happens If I'm Not Eligible?

If you're found to be ineligible, the study team will inform you that you don't meet the entry requirements. Any information previously collected will be deleted. If you have already consented to use the study app, your information will be deleted, and your account will be deactivated.

6. What are the possible benefits of taking part?

We cannot promise that you will benefit from taking part in the study, but the information gained may help us to treat present and future patients with chronic visceral pain.

7. What are the possible disadvantages and risks of taking part?

Pain tolerance testing is intended to be uncomfortable, but you can stop the procedure at any time.

The research team have been trained and are experienced in undertaking all the tests. The tests may cause temporary redness. The equipment used is calibrated and designed with in-built cut-off limits, particularly for people who cannot feel pain, so that the risk of skin injury is very low.

The wearable sensors are not intended for use as diagnostic monitor, or as a replacement for standard-of-care patient monitoring. The data being collected is only intended for use in research settings and is not able to be used for diagnosis or treatment.

The adhesive on the chest skin sensor may cause mild discomfort, skin irritation, redness, itching, rash, or contact dermatitis in some individuals. Wearable sensors should be removed if any pain or discomfort occurs. The adhesives are single-use only and should not be reused.

The chest skin sensors should not be used on patients with known allergies, or hypersensitivities to nickel, or those with implanted pacemakers or defibrillators. The wearable sensors are not MRI, CT scan or X-ray safe, and therefore should be removed prior to an MRI scan, CT scan or prior to any radiography.

Both chest skin and watch sensors **are not waterproof** and should not be worn in water or whilst showering or bathing.

8. Will my General Practitioner routinely be informed?

No, your GP will not be routinely informed of your participation in this study.

9. Expenses and payment

If you decide to participate in this study, you will be paid £15 per visit. This includes compensation for reasonable costs of travelling (to and from the study site) and parking

incurred by your participation. Details of how and when payments will be made will be available from the study team, who will follow their local policy. You will only be paid if you attend and complete the study visit.

Section 2: Study conduct

10. Will my taking part be kept confidential?

Transparency Statement under General Data Protection Regulation (GDPR)

Cambridge University Hospitals NHS Foundation Trust (CUH) and The University of Cambridge are the Sponsors for this study based in the United Kingdom. They will be using information from you and your medical records in order to undertake this study and will act as the data controller for this study. This means that they are responsible for looking after your information and using it properly. The Sponsor organisations will keep identifiable information about you for up to 1 year after the study has finished to ensure your safety and allow the study to be reviewed by the authorities after it is finished.

Your rights to access, change or move your information are limited, as the Sponsor organisations need to manage your information in specific ways in order for the research to be reliable and accurate. To safeguard your rights, we will use the minimum personally identifiable information possible.

You can find out more about how the Sponsors use your information using the information below:

- For Cambridge University Hospitals NHS Foundation Trust, please visit: https://www.cuh.nhs.uk/patient-privacy/ or email the Data Protection Officer at: cuh.gdpr@nhs.net

- For University of Cambridge, please visit:

https://www.medschl.cam.ac.uk/research/information-governance/, or email

the Information Governance team at: researchgovernance@medschl.cam.ac.uk

Cambridge University Hospitals will collect your name, NHS number and contact details to contact you about this study, and make sure that relevant information about the study is recorded for your care, and to oversee the quality of the study. Individuals from the Sponsors and regulatory organisations may look at your medical and research records to check the accuracy of this study. Cambridge University Hospitals will pass these details to the Sponsors along with the information collected from you and your medical records. The only people in the Sponsor organisations who will have access to information that identifies you will be people who need to contact you in relation to this study and to audit the data collection process. Cambridge University Hospitals will keep identifiable information about you from this study for up to 1 year after the study has finished.

All information collected about you as a result of your participation in the study will be kept strictly confidential. Your personal and medical information will be kept in a secured file and be treated in the strictest confidence.

The people who analyse the information will not be able to identify you and will not be able to find out your name, NHS number or contact details. Only anonymous study data, without any personal information will be published at the end of the study.

When you agree to take part in this study, the information about your health and care may be provided to researchers running other research studies in this organisation and in other organisations. These organisations may be universities, NHS organisations or companies involved in health and care research in this country or abroad. Your information will only be used by organisations and researchers to conduct research in accordance with the UK Policy Framework for Health and Social Care Research.

This information will not identify you and will not be combined with other information in a way that could identify you. The information will only be used for the purpose of health and care research and cannot be used to contact you or to affect your care. It will not be used to make decisions about future services available to you, such as insurance.

11. What will happen to my data collected during the study?

Your personal and study related data will be stored on secure servers at the University of Cambridge. Your personal data can only be accessed by members of the research team for the purpose of research.

Your personal data will be also stored on secure servers at Cambridge Digital Health. The app called 'ADVANTAGE Diary' has been designed by Cambridge Digital Health, to align with Data Security Standard 4 (equivalent requirement for hosting on NHS servers). The app will be managed via platform / server hosted within the UK and storage is compliant with GDPR and UK Data Protection Act 2018. Your personal data can only be accessed by members of the research team for the purpose of research.

Anonymised sensor data will be stored on secure servers at the United States, but no personal information will be stored outside of the United Kingdom.

We will never divulge or use your personal data without your expressed permission. We will only ever share fully anonymised material or data with other named researchers based in the NHS, universities or commercial companies including those who are based outside the United Kingdom.

12. What if I decide I no longer wish to participate in the study?

You are free to leave the study at any time without giving a reason, and without affecting your future care/medical treatment or legal rights. If you decide not to participate any further, no further study related tests will be performed on you, and no further research samples will be collected. However, any information/tests already collected, or results from tests already performed on you will continue to be used in the study analysis.

13. What if there is a problem?

Any complaint about the way you have been dealt with during the study or any possible harm you might suffer will be addressed. If you have any concerns about any aspect of this study, you should speak to your study team who will do their best to answer your questions.

In the event that something does go wrong, and you are harmed by taking part in the research and this is due to someone's negligence then you may have grounds for a legal action for

compensation against Cambridge University Hospitals NHS Foundation Trust. If your claim is successful, your legal costs will be met. The normal National Health Service complaints mechanisms will still be available to you (if appropriate). The University has obtained insurance which provides no fault compensation i.e., for non-negligent harm, you may be entitled to make a claim for this.

If you wish to complain or have any concerns about any aspect of the way you have been approached or treated during this study, you can do so through the NHS Complaints procedure. In the first instance it may be helpful to contact the Patient Advice and Liaison Service (PALS) on 01223 216756 or via email, cuh.pals@nhs.net.

14. What will happen to the results of the study?

The results of the study will be anonymous, and you will not be identified from any of the data produced. When the results of the study are available, they will be published in peer reviewed medical journals and used for medical presentations and conferences.

Anonymous coded data from the study will be made available to other researchers in line with national and international data transparency initiatives. These researchers may be outside the United Kingdom where privacy laws may not be as stringent however, none of your personal details will be shared outside of the research team.

15. Who is organising and funding the research?

The study has been organised and jointly sponsored by Cambridge University Hospitals NHS Foundation Trust and the University of Cambridge.

This study is being conducted as part of the UK Medical Research Council and Versus Arthritis Advanced Pain Discovery Platform (APDP), which is funded till 2026.

16. Who has reviewed the study?

Members of the patient and charities advisory board, clinicians, and scientists, as part of the ADVANTAGE visceral pain consortium, have reviewed all aspects of this study.

All research in the United Kingdom is looked at by an independent group of people called a Research Ethics Committee. A favourable ethical opinion has been obtained from London-Surrey Research Ethics Committee.

17. Contact details

If you have any questions or require further information about the study, please feel free to contact:

Study contact	Study role	Contact details
Jimena Teran Perez	Research Nurse	enquiries-pain@medschl.cam.ac.uk
Ully Gull	Research Nurse	enquiries-pain@medschl.cam.ac.uk
Dr Nicholas Shenker	Local Investigator	enquiries-pain@medschl.cam.ac.uk
Jason Crawte	Research Coordinator	enquiries-pain@medschl.cam.ac.uk
Professor Geoff Woods	Investigator	enquiries-pain@medschl.cam.ac.uk

Thank you for reading this information sheet and for considering taking part in this research study.

scan for link to send email

ADVANTAGE VISCERAL PAIN CONSORTIUM | UNIVERSITY OF CAMBRIDGE | NHS Cambridge University Hospitals NHS Foundation Trust

CONSENT FORM – Healthy Volunteers

Title of Project:	Clinical ADVANTAGE Study	Participant study ID:	
Name of Researcher:	Professor Geoff Woods		

Taking part	Please initial next to your answer to the statement	
1. I confirm that I have read the information sheet dated ddmmyy (version xx) for the above study. I have had the opportunity to consider the information, ask questions and have had those answered satisfactorily.	Yes	
	No	
2. I understand that my participation is voluntary and that I am free to withdraw at any time without giving any reason, without my medical care or legal rights being affected.	Yes	
	No	
3. I understand that relevant sections of my medical notes and data collected during the study, may be looked at by individuals from the Sponsor regulatory authorities or from the NHS organisations, where it is relevant to my taking part in this research. I give permission for these individuals to have access to my data and/or medical records.	Yes	
	No	
**Data**	**Please initial next to your answer to the statement**	
4. I agree that my personal and contact details will be stored on secure databases at the University of Cambridge and Cambridge Digital Health. These details will be stored for up to one year after the study has finished. They will only be used to communicate with me during the study.	Yes	
	No	
5. I agree that my anonymised data may be stored indefinitely. I understand that data from which I cannot be identified may be used in future research studies at Cambridge or other research institutions.	Yes	
	No	
6. I agree to my anonymised data being shared with other biomedical researchers conducting ethically approved studies, including those from other countries, commercial companies and those working on research beyond the field of chronic pain.	Yes	
	No	
**Wearables**	**Please initial next to your answer to the statement**	

7. I agree to use the wearable sensor(s) provided by the study team, as outlined in the participant information sheet.	Yes	
	No	
8. I agree to return the wearable sensor(s), the unused adhesives, device charger and supplied iPad to the research team.	Yes	
	No	
9. I agree to complete the mobile pain app as outlined in the participant information sheet.	Yes	
	No	
Please initial next to your answer to the statement		
6. I agree to take part in the above study.	Yes	
	No	

______________________________ ______________________ ______________________ _______

Name of Participant | Date | Signature

______________________________ ______________________ ______________________ _______

Name of Person seeking consent | Date | Signature

When completed: 1 for participant; 1 for researcher site file; 1 to be kept in medical notes.
